# Sleep quality and related factors among healthcare workers in designated quarantine hospital site in post-pandemic based on the health ecological model: a cross-sectional study in Nanjing, China

**DOI:** 10.3389/fpubh.2024.1419665

**Published:** 2024-07-04

**Authors:** Han Zhou, Lei Shen, Huafeng Tan, Jiefang Zhou, Qiyi Zheng, Dongdong Jiang

**Affiliations:** ^1^Nanjing Lishui District Hospital of Traditional Chinese Medicine, Nanjing, China; ^2^Zhenjiang Mental Health Center, Zhenjiang, Jiangsu, China; ^3^School of Public Health, Zhejiang University School of Medicine, Hangzhou, China

**Keywords:** sleep quality, healthcare workers, designated quarantine hospital site, health ecological model, cross-sectional study

## Abstract

**Aims:**

This study aims to assess the status and related factors among healthcare workers (HCWs) in designated quarantine-hospital-site (DQHS) based on the model of health ecology.

**Methods:**

The cross-sectional study was conducted from April to May, 2022, which included 351 valid samples. We measured sleep quality using the Pittsburgh Sleep Quality Index, which encompasses seven dimensions: subjective sleep quality, sleep latency, sleep duration, habitual sleep efficiency, sleep disturbances, use of sleep medication, and daytime dysfunction. Each dimension is scored individually, contributing to an overall sleep quality score. Factors associated with the sleep quality of HCWs in DQHS were divided into individual, behavioral, interpersonal and social dimensions. Hierarchical linear regressions were conducted to identify the potential factors associated with sleep quality among HCWs in DQHS.

**Results:**

HCWs in DQHS had a statistically higher sleep quality than the Chinese national norm. HCWs who were female, afraid of Coronavirus disease, had more negative emotions, frequently worked overtime, were married, and had a higher income were more likely to experience worse sleep quality (*p* < 0.05), while those who worked between 51 and 70 h weekly, treated over 10 patients daily, and engaged in more health behaviors may have better sleep quality (*p* < 0.05).

**Conclusion:**

This study revealed a worrying level of sleep quality among HCWs in DQHS. The government, hospital managers, and families should collaborate to ensure the sleep quality of HCWs in DQHS.

## Introduction

Sleep is essential to human health, acting as a restorative neurobehavioral state (1). Studies have shown that subpar sleep quality is linked to serious health issues like cardiovascular disease (2), obesity (3), and mental health (4). While 9 to 45% of the general population experiences poor sleep (5), healthcare workers (HCWs) face it more often due to demanding schedules and shift work (6). This can impair their performance and increase errors, leading to emotional issues, daytime dysfunction, and higher absenteeism and turnover rates. Ultimately, this affects patient care, potentially resulting in dissatisfaction due to the reduced quality of service (7–9).

Since COVID-19’s onset, Chinese HCWs have significantly contributed to the country’s relatively low death rate (8.12 per 10,000 people) (10). Tasked with key roles in prevention and control, these workers face physical and mental fatigue due to an overburdened health system, insufficient protective gear, and a high risk of infection (11). Consequently, they are more prone to sleep disorders (12)—with research indicating that over half of frontline HCWs have experienced such issues (13, 14). Even after easing quarantine measures, about 41% of HCWs still reported sleep disorders (11). Therefore, addressing the factors impacting their sleep and enhancing sleep quality are critical concerns.

Previous studies have shown that sleep quality has a significant impact on health (15, 16), especially among HCWs (17, 18). Poorer sleep quality may lead to lower work productivity (19), more medical errors (20) and burnout (21) for HCWs. Research has identified that both personal (such as education and socio-economic status) (22, 23) and work-related factors (like job type and workload) (24) influence HCWs’ sleep quality. Recent studies also highlight the impact of personal emotions (25), fear of COVID-19 (26), isolation conditions (13), and health behaviors (27). Despite the extensive research on factors affecting HCWs’ sleep during the COVID-19 pandemic, there’s limited focus on those working in designated quarantine-hospital-sites (DQHS) post-pandemic. Addressing this gap is crucial for targeted interventions. Notably, there are many ways to measure sleep quality in empirical research, including scale measurements (such as the Pittsburgh Sleep Quality Index), sleep diaries, sleep activity recorders (such as electronic wristbands), and so on. The advantage of the scale is that it is easy to manage and can quickly collect a large amount of data, but there may be recall bias due to participants’ self-reports (28); A sleep diary can provide detailed sleep information, but it requires high cooperation and accurate recording from the subjects (29); while the sleep activity recorder is not affected by supervisor’s reports and can be continuously monitored for a long time, but it is costly and requires professional personnel to perform data analysis (30).

**Figure 1:**
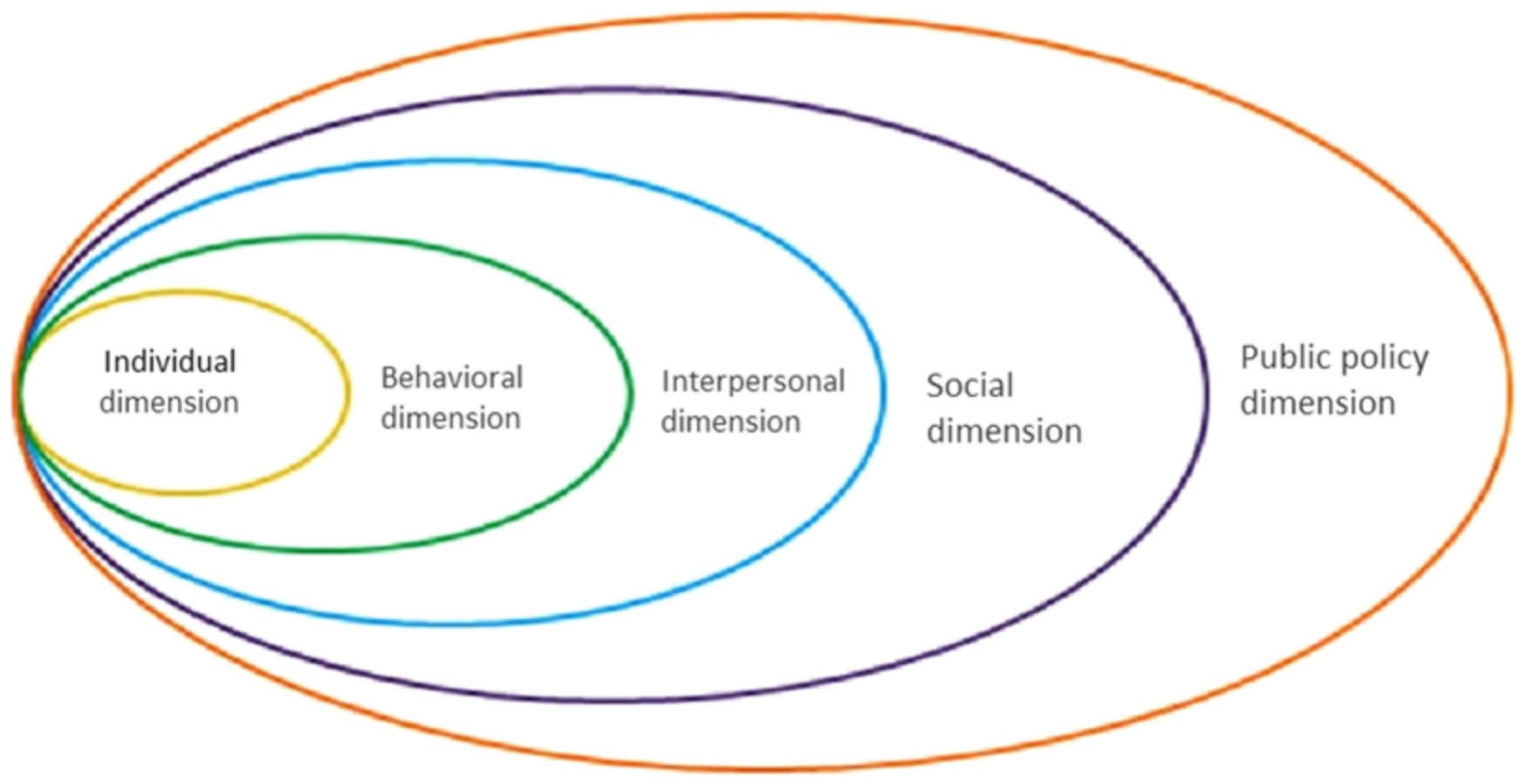
Health ecological model.

With COVID-19 more controlled, China’s easing of restrictions has not removed the threat entirely, as sporadic outbreaks persist. Specifically, after the large-scale control of the epidemic on December 11, 2021, China began implementing a “dynamic COVID-zero strategy” (31). To prevent a resurgence, special quarantine points have been established for isolation. HCWs at these Designated Quarantine Hospital Sites (DQHS) continue to treat and monitor infected individuals. Their extended on-site work with limited home visits means their sleep quality is a pressing concern. This study aims to explore the sleep quality status of the HCWs in DQHS and to identify their potential correlation based on the model of health ecology (HEM). The HEM has proven that personal attributes, family factors, and social support can affect an individual’s health (32). This model is based on an inside-out method and contains 5 dimensions (the theoretical framework is shown in Figure 1), including individual, behavioral, interpersonal, social and public policy dimension (33). Previous studies have proven the main advantage of the health ecology model lies in its comprehensiveness and multi-level perspective, which makes it a powerful tool for understanding and improving individual and group health behaviors (34, 35). Studies that have used HEM primarily concentrate on older individuals (36, 37) and patients (32, 38), with minimal focus on HCWs. Therefore, the HEM can also help us understand the complexity of sleep problems among healthcare workers from multiple perspectives and provide us with a structured approach to designing and implementing effective intervention strategies. There is limited literature studying healthcare workers’ sleep quality, especially those HCWs in DQHS. Our study aims to identify factors that affect their sleep quality and gather baseline data to inform effective health-promotion programs by local and national health authorities. Ultimately, we aim to spotlight the needs of DQHS HCWs to the public and provide a reference on enhancing sleep quality in future quarantine situations.

## Methods

### Contexts and samples

To bolster the capabilities of COVID-19 treatment centers, Jiangsu Province’s Health Commission mandates that hospitals with robust overall capabilities, high-quality treatment, and a solid base for infection control be designated for COVID-19 care. In Nanjing, three such hospitals have been chosen, where HCWs and patients are kept in isolation—these are the DQHS. HCWs there have to remain on-site for extended periods to prevent carrying the virus outside and risking societal transmission, unlike their non-quarantined counterparts.

Given the similarity in scale, comprehensive strength, and number of HCWs among the three DQHSs in Jiangsu province, this study employed the random sampling principle to investigate all HCWs in a single hospital. In this DQHS, HCWs come from two tertiary hospitals and eight community hospitals, respectively. During the period from December 2021 to December 2022, China was implementing the policy of “dynamic COVID-zero strategy” for COVID-19. We conducted this cross-sectional study from April to May 2022. We informed all the participants on the title page of the questionnaire that the survey was anonymous and that the questionnaire would not include their personal or private information. All the questions were completed voluntarily by the respondents themselves. Participants in this study had the following inclusion criteria: (1) having worked or lived at DQHS for more than 1 month; (2) voluntarily participating in this survey; The exclusion criteria were: (1) non-HCWs (such as cleaners and cafeteria staff); (2) individuals who have been diagnosed with severe mental illness. We sent a total of 390 questionnaires and received 370 questionnaires. We have set an attention detection question in the online questionnaire (“Please choose the third option for this question”) to ensure that participants answer the questions carefully. In the final collected questionnaire, everyone passed the attention detection question. In addition, to ensure the data quality, we conducted a manual logic error-check inference and excluded 11 questionnaires because of consistent answers or large areas of blank answers. Therefore, there were 359 questionnaires included finally, with an effective rate of 97.03%.

### Measures

#### The composition of HEM

According to the HEM theory and considering that it is challenging to quantify macro-systemic policy environment indicators, we only construct the theoretical framework (Table 1) of this study from four dimensions: individual, behavioral, interpersonal and social. We confirm the indicated variables in each dimension based on previous studies (32, 34, 36–40).

**Table 1:** Variables related to sleep quality among HCWs.

Layers	Variables
Individual dimension	Sex
	Age
	Coronavirus disease fear
	Positive emotions
	Negative emotions
Behavioral dimension	Average working hours per week
	Patients treated per day
	Work overtime
	Health behavior score
Interpersonal dimension	Marital status
	Perceived social support
Social dimension	Education level
	Income
	Classification of occupations
	Job title

(1) The individual dimension included five indicators (sex, age, coronavirus disease fear, positive emotions and negative emotions). Coronavirus disease fear was measured by the fear of the COVID-19 scale (FCV-19), which was developed by Ahorsu et al. (41). The scale has 7 items, and the higher the FCV-19 score of the respondents means the respondent has higher level of Coronavirus disease fear. The Cronbach α of FCV-19 in this study was 0.910. The Positive and Negative Affect Schedule (PANAS) (42) assessed both positive and negative emotions. PANAS contains of 20 questions, with 10 questions measuring positive emotions and 10 questions measuring negative emotions. In this study, the Cronbach α of PANAS was 0.909. (2) The behavioral dimension consisted of four indicators (average working hours per week, patients treated per day, work overtime and health behavior). Average working hours per week, patients treated per day, and work overtime were grouped into 3 classes (≤50 h, 51 h ~ 70 h, and ≥ 71 h; 0–10, 11–30, and ≥ 31; never, sometimes, and frequently), respectively, Health behavior was assessed by the Chinese version of the Health-Promoting Lifestyle Profile (HPLP-C) (43). The HPLP-C is comprised of 6 parts (self-realization, health responsibility, physical activity, nutrition, interpersonal relations, and stress management), for a total of 42 items. In this study, we use the total score to represent the health behaviors. The HPLP-C score ranges from 42 to 168, and a higher score indicates better health behaviors. The Cronbach α of HPLP-C in this study was 0.977.

(3) The interpersonal dimension included two indicators (marital status and perceived social support). Marital status was divided into two groups (married and not married), while perceived social support was measured by a multidimensional scale of perceived social support (MSPSS) (44). MSPSS was used to measure the degree of support that the individual perceives from various sources of social support inside and outside the family. The total score of MSPSS reflects the level of overall social support that the individual perceived. The MSPSS has 12 items and uses a 7-level scoring system with a score range of 12–84. The Cronbach α of MSPSS in this study was 0.968.

(4) The social dimension included four indicators (education level, income, classification of occupations, and job title). Education level, income, classification of occupations and job title were all grouped into 3 categories (< college, college, and > post-graduate; ≤60,000 Chinese Yuan, 60,001 ~ 120,000 Chinese Yuan, and ≥ 120,001 Chinese Yuan; clinical medicine, nursing, and others).

#### The measurement of sleep quality

We adopted the Pittsburgh Sleep Quality Index (PSQI) to measure the sleep quality of HCWs in DQHS (28). The PSQI has 19 items that contain 7 dimensions (subjective sleep quality, sleep latency, sleep duration, habitual sleep efficiency, sleep disturbance, use of sleeping medication, and daytime dysfunction). Subjective sleep quality reflects an individual’s overall satisfaction with sleep; sleep latency indicates the duration from getting into bed to the onset of sleep; sleep duration measures the total length of night-time sleep; habitual sleep efficiency represents the ratio of actual sleep time to total time in bed, reflecting sleep continuity; sleep disturbances assess the frequency of nocturnal awakenings and related symptoms; use of sleep medications indicates the degree of dependence on pharmacological aids; and daytime dysfunction evaluates the limitations in daily activities due to sleep issues. Together, these dimensions provide a comprehensive, multifaceted evaluation of sleep quality. Many scholars have proved the great reliability and validity of the PSQI (45, 46). The higher score on PSQI represents poorer sleep quality. The Cronbach α of PSQI in this study was 0.851.

### Statistical analysis

All analyses were performed in Statistical Package for the Social Sciences (SPSS) version 26.0 for Windows (SPSS Inc., Chicago, IL, United States), with a significance level of 0.05 (two-tailed).

Categorical variables in the study were described using frequency and proportion, and continuous variables by mean and standard deviation (SD). We calculated the sleep quality score for each categorical group using the mean ± SD and employed Pearson correlation to assess the relationships between sleep quality and continuous variables. Moreover, the average scores of all dimensions of the PSQI were compared with the Chinese national norm (47) through *t*-tests. We explored unadjusted associations between demographic features and total PSQI score using t/F tests or Pearson correlation. Lastly, we used hierarchical linear regression analysis to model factors related to HCWs’ sleep quality. In each model (1–4), we added variables related to HEM dimensions. We assessed collinearity, and the results showed tolerance values ranging from 0.361 to 0.901 (all above 0.1) and VIF values from 1.110 to 2.771 (all below 5), indicating no issues with collinearity among the variables.

### Ethical statement

The Institutional Review Board of Nanjing Lishui District Hospital of Traditional Chinese Medicine approved this research (2022LCGC001), and all methods were conducted in accordance with the relevant guidelines and regulations. All questionnaires had consent information, which informed participants that the survey was optional and data would be collected only for statistical analysis. After consenting to this information, all of the participants completed the questionnaires voluntarily.

## Results

### The distribution of demographic characteristics and sleep quality scores of the samples

Table 2 displays the demographic characteristics of the samples. Among the samples, 68.25% were female and the average values of age, coronavirus disease fear, positive emotions, negative emotions, healthy behaviors, and perceived social support were 30.69, 17.56, 29.90, 22.28, 117.66, and 63.03, respectively. Most HCWs (78.83%) worked less than 50 h and below weekly, and most of them (39.00%) treated less than 10 patients daily. Nearly 80% of the HCWs have worked overtime. Over half of them were married, and most of them received college degrees. In our study, 65.46% of HCWs earned between 60,001 and 120,000 Chinese Yuan, and 57.66% of the individuals work as nurses, with 61.28% holding an elementary and below job title.

**Table 2:** Demographic characteristics and scores of sleep quality among the study samples.

Layers	Variables	Categories	N (%)/Mean ± SD	Mean ± SD/ Correlation	*p* value
Individual layer	Sex	Male	114 (31.75)	5.66 ± 3.14	<0.001
		Female	245 (68.25)	7.29 ± 3.27	
	Age	–	30.69 ± 7.24	0.010	0.846
	Coronavirus disease fear	–	17.56 ± 6.15	0.335	<0.001
	Positive emotions	–	29.90 ± 7.95	−0.240	<0.001
	Negative emotions	–	22.28 ± 7.67	0.234	<0.001
Behavioral layer	Average working hours per week	≤50 h	283 (78.83)	6.80 ± 3.46	0.074
		51 h ~ 70 h	62 (17.27)	6.27 ± 2.82	
		≥71 h	14 (3.90)	8.50 ± 4.73	
	Patients treated per day	0–10	140 (39.00)	7.01 ± 3.13	0.309
		11–30	136 (37.88)	6.81 ± 3.34	
		≥31	83 (23.12)	6.31 ± 3.56	
	Working overtime	Never	74 (20.61)	5.94 ± 3.54	<0.001
		Sometimes	200 (55.71)	6.48 ± 3.09	
		Frequently	85 (23.68)	8.20 ± 3.24	
	Healthy behavior score	-	117.66 ± 24.77	−0.226	<0.001
Interpersonal layer	Marital status	Single	167 (46.52)	6.52 ± 3.38	0.173
		Married	192 (53.48)	7.00 ± 3.25	
	Perceived social support	–	63.03 ± 13.10	−0.157	0.003
Social layer	Educational level	< College	108 (30.08)	6.58 ± 3.21	0.002
		College	192 (53.48)	7.26 ± 3.34	
		≥Post-graduate	59 (16.43)	5.55 ± 3.14	
	Income	≤60,000	63(17.55)	6.31 ± 3.54	0.147
		60,001 ~ 120,000	235 (65.46)	6.72 ± 3.28	
		≥120,001	61 (16.99)	7.45 ± 3.16	
	Occupation	Physician	124 (34.54)	5.96 ± 3.21	<0.001
		Nurse	207 (57.66)	7.36 ± 3.39	
		Others	28 (7.80)	6.03 ± 2.28	
	Job title	Elementary and below	220 (61.28)	6.68 ± 3.20	0.721
		Intermediate	116 (32.31)	6.98 ± 3.63	
		Advanced	23 (6.41)	6.65 ± 2.82	

Single, including unmarried, divorced and widowed; SD, standard deviation; *N* (%), number (proportion).This table applied the statistical methods of *t*-test and Pearson correlation test.

In this study, the results also showed the statistical significance between sleep quality and sex, coronavirus disease fear, positive emotions, negative emotions, working overtime, healthy behaviors, perceived social support, educational level, and occupation (*p* < 0.05).

### Comparison of HCWs’ sleep quality with the national norm

Table 3 records the scores of sleep quality among HCWs at DQHS. It reveals that all seven dimensions (subjective sleep quality, sleep latency, sleep duration, habitual sleep efficiency, sleep disturbance, use of sleeping medication, and daytime dysfunction) for HCWs in DQHS are significantly higher than the Chinese national norm. The total score for HCWs in DQHS was 6.77 ± 3.32.

**Table 3:** Assessment results of each dimension of PSQI.

Dimensions	Reference Range of China (Mean ± SD)	Participants’ Score in this Study (Mean ± SD)	*t*	*p* value
Subjective sleep quality	0.63 ± 0.68	1.23 ± 0.70	16.449	<0.001
Sleep latency	0.70 ± 0.86	1.48 ± 0.91	16.416	<0.001
Sleep duration	0.70 ± 0.58	1.22 ± 0.82	12.247	<0.001
Habitual sleep efficiency	0.15 ± 0.47	0.47 ± 0.82	7.527	<0.001
Sleep disturbance	0.90 ± 0.44	1.07 ± 0.55	5.745	<0.001
Use of sleeping medication	0.06 ± 0.24	0.11 ± 0.46	1.990	0.047
Daytime dysfunction	0.73 ± 0.83	1.18 ± 0.91	9.252	<0.001
Total score	3.88 ± 2.52	6.77 ± 3.32	16.530	<0.001

SD, standard deviation. This table applied the statistical methods of *t*-test.

### Hierarchical linear regression analysis on the sleep quality of HCWs in DQHS

We selected sleep quality as the dependent variable and, step by step, used the variables of individual, behavioral, interpersonal and social dimensions according to the HEM (model 1 to model 4). In the final model (model 4), we found that HCWs in DQHS who were female (*β* = 1.220, *p* = 0.007), had a higher level of coronavirus disease fear (*β* = 0.114, *p* < 0.001), had negative emotions (*β* = 0.084, p < 0.001), frequently worked overtime (*β* = 1.182, p < 0.001), were married (*β* = 1.154, *p* = 0.006), and had a monthly income over 120,000 (*β* = 1.523, *p* = 0.016) received poorer sleep quality. While those HCWs who worked hours between 51 and 70 h (*β* = −1.008, *p* = 0.032), treated patients between 11 and 30 (*β* = −1.026, *p* = 0.007) and over 30 (β = −1.183, p = 0.006), engaged in more health behaviors (*β* = −0.022, *p* = 0.031), had better sleep quality (Table 4).

**Table 4:** The association between demographic characteristics and the total score of PSQI among medical staff.

Layers	Variables	Categories	Model 1		Model 2		Model 3		Model 4	
			*β*	*p* value	*β*	*p* value	*β*	*p* value	*β*	*p* value
Individual layer	Sex (ref = male)	Female	1.325	<0.001	1.288	<0.001	1.386	<0.001	1.220	0.007
	Age	–	0.067	0.005	0.065	0.008	0.028	0.329	0.024	0.476
	Coronavirus disease fear	–	0.127	<0.001	0.111	<0.001	0.114	<0.001	0.114	<0.001
	Positive emotions	–	−0.073	0.001	−0.053	0.080	−0.057	0.059	−0.051	0.100
	Negative emotions	–	0.090	<0.001	0.079	0.001	0.091	<0.001	0.084	<0.001
Behavioral layer	Average working hours per week (ref = 50 h and lower)	51 ~ 70 h			−1.146	0.012	−1.083	0.016	−1.008	0.032
		71 h and higher			0.076	0.928	0.104	0.900	0.283	0.735
	Patients treated per day (ref = 0–10)	11–30			−0.870	0.018	−1.019	0.005	−1.026	0.007
		31 and higher			−1.000	0.016	−1.173	0.005	−1.183	0.006
	Work overtime (ref = Never)	Sometimes			0.346	0.406	0.368	0.370	0.254	0.541
		Frequently			1.842	<0.001	1.867	<0.001	1.182	<0.001
	Health behavior score	–			−0.011	0.251	−0.020	0.049	−0.022	0.031
Interpersonal layer	Marital status (ref = single)	Married					1.116	0.004	1.154	0.006
	Perceived social support	–					0.025	0.107	0.020	0.203
Social layer	Education level (ref = Under college)	College							−0.246	0.579
		≥Post-graduate							−0.734	0.253
	Income (ref = ≤60,000)	60,001 ~ 120,000							0.334	0.478
		120,001 and higher							1.523	0.016
	Occupation category (ref = Physician)	Nurse							0.149	0.770
		Others							−0.016	0.984
	Job title (ref = Elementary and below)	Intermediate							−0.188	0.682
		Advanced							−0.246	0.771
*R* ^2^			0.206		0.268		0.295		0.315	

Ref, reference group. This table applied the statistical methods of hierarchical linear regression analysis.

## Discussion

To our knowledge, this is the first study focusing on the sleep quality of HCWs in the DQHS, especially during the implementation of “dynamic COVID-zero strategy” in China. In this cross-sectional study, we found that the total sleep quality and seven dimensions of PSQI among HCWs in the DQHS were utterly different from the Chinese national norm. Specifically, HCWs in the DQHS were more likely to suffer from conditions of low subjective sleep quality, prolonged sleep latency, less sleep duration, low habitual sleep efficiency, sleep disturbance, frequent use of sleeping medication and daytime dysfunction, because the scores of these seven dimensions were statistical significantly higher than the Chinese national norm. According to Ahrberg et al. (48), the total score of PSQI above 5 is a reliable and validated indicator for clinically relevant pathological sleep. Thus, the results (6.77 ± 3.32 > 5) of our study indicated worrying circumstances with regard to the sleep quality and symptoms of sleep-related factors among HCWs in the DQHS. We speculate that the unique environment and job responsibilities of HCWs in the DQHS, which include frequent emergencies, high work intensity, and mental stress from COVID-19 fears, may contribute to their sleep issues. Several systematic reviews and meta-analysis (49–51) have suggested that prolonged lockdown and quarantine can cause anxiety, depression, irritability, and other psychological disturbances, which are known to disrupt sleep (52). The study underscores the need for increased attention from both government bodies and hospital management to the sleep health of DQHS HCWs to mitigate the risks associated with their challenging work conditions.

In line with previous studies, females were more likely to experience poor sleep quality than males (51, 52). Understandably, females were more susceptible to negative information than males, increasing anxiety and leading to a decrease in sleep quality (53). Even doctors may be afraid of the sudden COVID-19 pandemic, due to its strong infectivity (54). In this study, fear of coronavirus disease made HCWs more likely to suffer from bad sleep quality. Research has proven that fear of Coronavirus disease will cause anxiety, depression, and stress in HCWs (55, 56), which may lead to poor sleep quality. Moreover, our study also found that HCWs with more negative emotions may experience poorer sleep quality, consistent with Pérez-Fuentes et al.’s study (57). Considering that emotions can spread interpersonally (58), it is urgent to pay attention to the emotional changes of HCWs in the DQHS.

Our study presents an unexpected finding: HCWs in DQHS who averaged 51–70 working hours per week and treated more patients daily enjoyed better sleep quality compared to those working fewer than 50 h and treating less than 10 patients daily. Typically, there is a negative correlation between work hours and sleep quality (59, 60), and seeing more patients also means an extension of working hours. We hypothesise that those with longer hours may have adapted to the demanding rhythm, thus establishing a stable physiological routine due to the necessity of staying on-site at isolation facilities. Alternatively, the exhaustion from an overload of work could paradoxically enhance sleep quality during limited rest periods. However, these findings could be subject to ecological fallacies, and further investigation with a larger sample size is required for validation. In agreement with previous studies (61, 62), HCW in the DQHS working overtime frequently had poorer sleep quality. Our study also confirms that good sleep quality appears to be dependent on healthy behaviors, similar to other studies (27, 63). Thus, it is suggested to encourage HCWs in the DQHS to arrange working hours and conduct more health behaviors reasonably. For example, DOHS can provide HCWs with fitness equipment, nutritious food, and so on.

Research indicates a positive link between robust social networks and good sleep quality (64, 65). In our study, married HCWs in the DQHS were more inclined to suffer from poorer sleep quality than single ones. This could stem from worry over potentially infecting their families (66), or from the struggle to balance work and family obligations, leading to stress and sleep disturbances (23). It is noted that there was no significant difference between perceived social support and sleep quality among HCWs in the DQHS in our study. We speculate that the isolating environment and demanding workloads might diminish the beneficial impact of social support on sleep (67, 68). Given these findings, enhancing welfare benefits for HCWs in DQHS is crucial to alleviate family-related concerns and bolster social support.

Existing research has found that lower socioeconomic status is negatively correlated with poorer sleep quality (69), education level is more positively correlated with sleep quality (70), and job titles are positively correlated with sleep quality (71). However, in our study, we only found that HCWs in the DQHS with higher incomes had worse sleep quality than those with incomes lower than 60,000. In general, HCWs with higher incomes have a higher chance of treating more severe patients and would be asked to address more serious problems. These may trigger higher pressure for them, which may lead to worse sleep quality. Therefore, the hospital managers should pay more attention to HCWs in the DQHS with higher incomes.

Our study’s findings may have some implications for health department administrators, hospital managers, and researchers conducting policy-making and interventions for those HCWs in the DQHS. Although COVID-19 has basically been controlled, these implications can also offer experience for staff in quarantine sites, such as HCWs in prison (72), HCWs in psychiatric hospitals (73), etc. Firstly, the government needs to establish an emergency service system to ensure the circulation of various resources at isolation points; Secondly, hospital managers should prepare contingency plans, especially psychological interventions for those HCWs, to prevent the spread of negative emotions; Thirdly, third-party resources can be introduced to support those HCWs; Fourthly, strengthen the psychological crisis intervention training for medical personnel and closely monitor their sleep quality.

Given the limited literature in China focusing on the sleep quality of HCWs in the DQHS, only a few studies could be referred to. It is noted that there are several limitations to this study. First, it is a cross-sectional study, which means the causal relationship between variables and the sleep quality of HCWs in the DQHS can not be inferred. This study only displayed the current status of this special population. Second, this study used a self-reported questionnaire, leading to a certain recall bias. Third, our investigation was limited to samples from a single DQHS in Nanjing. The representativeness of samples is limited. Therefore, more sample size and longitudinal or experimental designs should be further conducted. Fourth, our study has not collected balanced sample distribution data, which may lead to a lack of generalizability and interpretation of research findings. Future studies will be needed to solve the problem of an unbalanced sample distribution. Fifth, in this study, we only included the first four dimensions and did not address the public policy dimension due to its complexity (the difficulty in quantifying macro system policy environment indicators) and diversity. Further exploration of the impact of the public policy dimension on health can be conducted as resources permit. Sixth, according to the theory of health ecology (33), it originated from ecological and social ecological models, so it does indeed have a nested structure. Therefore, future research may consider using Hierarchical Linear Modeling (HLM) to further discover nested effects that affect the sleep quality of HCWs in sufficiently large sample sizes.

## Conclusion

As far as we know, this study is the first study focusing on the sleep quality status and its related factors of HCWs in the DQHS based on HEM during the implementation of dynamic COVID-zero strategy in China. This study demonstrated that the sleep quality of HCWs in the DQHS was statistically higher than the Chinese national norm. The study also showed that sex, fear of coronavirus disease, negative emotions, average working hours per week, patients treated per day, working overtime, health behaviors, marital status, income were the potential predictors of sleep quality among HCWs in the DQHS. Joint efforts (both health resource and social support) from the government, hospital managers, families, should be taken to assure the sleep quality of HCWs in the DQHS.

## Data availability statement

The original contributions presented in the study are included in the article/supplementary material, further inquiries can be directed to the corresponding author.

## Ethics statement

The studies involving humans were approved by The Institutional Review Board of Nanjing Lishui District Hospital of Traditional Chinese Medicine approved this research (2022LCGC001), and all methods were conducted in accordance with the relevant guidelines and regulations. The studies were conducted in accordance with the local legislation and institutional requirements. The participants provided their written informed consent to participate in this study.

## Author contributions

HZ: Data curation, Formal analysis, Investigation, Methodology, Writing – original draft, Writing – review & editing. LS: Conceptualization, Data curation, Formal analysis, Writing – original draft, Writing – review & editing. HT: Data curation, Formal analysis, Investigation, Methodology, Writing – review & editing. JZ: Data curation, Formal analysis, Investigation, Methodology, Writing – review & editing. QZ: Data curation, Formal analysis, Writing – original draft, Writing – review & editing. DJ: Conceptualization, Data curation, Supervision, Validation, Writing – review & editing.
